# Supplementary material for: Hot super-Earths stripped by their host stars
Source: Nat Commun. 2016 Apr 11;7:11201. doi: 10.1038/ncomms11201 (PMC4831017; doi:10.1038/ncomms11201)
Supplement: Supplementary Information — Supplementary Table 1 and Supplementary References. [file ncomms11201-s1.pdf]

Supplementary Information: Hot super-Earths stripped by their host stars , M. S. Lundkvist et al.

Supplementary Table 1: Parameters for the 102 host stars with detected oscillations.

KIC number	$\Delta\nu$ (μHz)	σ($\Delta\nu$) (μHz)	T_{eff} (K)	σ(T_{eff}) (K)	[Fe/H] (dex)	σ([Fe/H]) (dex)	Ref.
3102384	124.7	0.4	5739	75	0.35	0.1	1
3425851	93.0	0.5	6343	85	-0.04	0.1	1
3531558	86.8	0.3	5747	85	0.03	0.1	1
3544595	145.8	0.4	5669	75	-0.18	0.1	1
3632418	60.9	0.6	6305	50	-0.03	0.1	2
3640905	30.7	0.5	4991	75	0.28	0.1	1
4141376	128.2	0.4	6134	91	-0.24	0.1	1
4143755	77.1	0.3	5622	106	-0.40	0.11	1
4262040	40.8	0.3	5299	147	-0.35	0.3	3
4349452	98.5	0.9	6270	79	-0.04	0.1	1
4450844	72.8	0.4	5968	59	-0.20	0.3	4
4769799	15.3	0.3	5079	143	-0.43	0.3	3
4815520	136.0	0.3	5755	50	0.20	0.08	5
4914423	82.0	0.4	5845	88	0.07	0.11	1
5042210	66.7	0.6	6007	120	0.05	0.15	3
5088536	110.0	0.4	5884	75	0.00	0.25	1
5094751	90.7	0.4	5952	75	-0.08	0.1	1
5383248	149.4	0.5	5690	77	0.04	0.07	6
5511081	63.6	0.4	5923	77	-0.07	0.1	1
5513648	76.9	0.3	5904	85	-0.07	0.1	1
5514383	113.3	0.6	6184	81	0.12	0.1	1
5561278	56.8	0.6	6081	75	-0.03	0.1	1
5652983	29.4	0.3	5198	95	0.19	0.11	1
5780885	56.4	0.7	6027	75	0.10	0.1	1
5866724	89.6	0.6	6169	50	0.09	0.08	1
5905822	82.2	0.4	6115	165	-0.06	0.3	3
6196457	67.1	0.5	5871	94	0.17	0.11	1
6268648	87.9	0.6	6044	117	-0.24	0.11	1
6278762	179.6	0.5	5046	74	-0.37	0.09	2
6448890	17.4	0.3	4840	97	0.20	0.16	1
6521045	77.1	0.5	5825	75	0.02	0.1	1
6528464	75.4	0.4	5588	99	-0.10	0.1	1

6678383	56.5	0.4	5711	74	-0.55	0.07	7
7199397	38.7	0.4	5896	75	-0.17	0.1	1
7215603	83.4	0.5	6173	93	0.17	0.1	1
7449136	77.2	0.4	6099	75	0.04	0.1	1
7582689	70.0	0.6	6004	75	0.07	0.1	8
7670943	88.3	0.6	6463	110	0.09	0.11	1
7887791	156.1	0.6	5547	100	-0.04	0.15	8
7941200	130.1	0.5	5952	119	0.02	0.15	3
8013439	86.0	0.4	6152	100	-0.18	0.1	7
8077137	69.0	0.7	6072	75	-0.09	0.1	1
8176564	77.7	0.4	6109	51	-0.20	0.3	4
8292840	93.2	0.6	6239	94	-0.14	0.1	1
8349582	83.4	0.4	5699	74	0.30	0.1	1
8478994	178.3	0.6	5417	75	-0.32	0.07	1
8494142	62.2	0.6	6144	106	0.13	0.1	1
8554498	62.2	0.4	5753	75	0.05	0.1	1
8684730	52.4	0.5	5882	87	0.16	0.1	1
8686097	103.7	0.3	5833	50	-0.27	0.08	5
8700771	69.0	0.4	5665	50	-0.39	0.08	5
8753657	121.0	0.4	5538	74	0.18	0.07	7
8801316	57.7	0.5	6718	183	-0.06	0.3	3
8803882	25.2	0.4	5250	169	0.06	0.3	3
8822366	71.1	0.3	6034	92	-0.14	0.1	1
8866102	94.5	0.7	6325	75	0.01	0.1	1
8868481	40.4	0.3	5779	67	-0.20	0.3	4
9072639	85.2	0.3	6306	220	-0.36	0.3	3
9394953	65.2	0.5	6018	65	-0.20	0.3	4
9414417	59.6	0.8	6253	75	-0.13	0.1	1
9451706	95.3	0.3	6106	106	0.33	0.1	1
9579208	61.0	0.6	6422	56	-0.20	0.3	4
9579641	131.2	0.3	6395	184	-0.22	0.3	3
9590976	72.3	0.4	6171	109	-0.12	0.1	7
9592705	53.4	0.5	6174	92	0.22	0.1	1
9602613	129.6	0.4	5467	148	-0.12	0.3	3
9696358	50.7	0.6	6131	75	0.13	0.1	1
9697131	59.5	0.4	6491	69	-0.20	0.3	4
9872292	65.0	0.7	6260	116	0.10	0.11	1

9955598	153.1	0.4	5460	75	0.08	0.1	1
10026544	26.0	0.6	6149	122	-0.42	0.15	3
10130039	143.5	0.4	5828	100	-0.08	0.1	7
10136549	55.9	0.4	5684	175	0.02	0.1	7
10264660	54.4	0.5	6378	75	-0.02	0.1	1
10272858	22.7	0.3	5208	68	-0.20	0.3	4
10289119	37.2	0.6	6056	165	-0.17	0.3	3
10585852	23.3	0.3	5048	75	-0.03	0.1	1
10586004	69.7	0.4	5770	83	0.29	0.1	1
10593626	165.2	0.4	5570	50	-0.26	0.06	K15
10666592	59.4	0.6	6350	80	0.26	0.08	1
10748390	186.6	0.4	4780	50	0.31	0.05	9
10794087	58.0	0.4	5721	50	0.25	0.08	5
10864656	18.6	0.5	4995	78	-0.07	0.1	1
10875245	86.4	0.4	5851	75	0.27	0.1	1
10963065	103.2	0.6	6104	74	-0.20	0.1	1
11127479	91.6	0.6	5998	57	-0.20	0.3	4
11133306	107.5	0.5	5982	82	-0.02	0.1	1
11250587	74.0	0.6	5862	97	0.27	0.11	1
11295426	101.5	0.4	5793	74	0.12	0.07	1
11337566	49.6	0.5	6467	173	-0.10	0.3	3
11401755	67.8	0.5	5911	66	-0.20	0.06	1
11446443	141.2	0.4	5850	50	-0.15	0.1	1
11512246	73.4	0.3	5828	100	-0.05	0.1	1
11600889	131.8	0.5	5476	75	0.33	0.07	6
11623629	161.1	0.4	5564	50	-0.14	0.08	5
11758544	78.2	0.3	6287	169	-0.27	0.3	3
11807274	74.9	0.7	6225	75	0.00	0.08	1
11853905	74.4	0.3	5781	76	0.09	0.1	1
11904151	117.9	0.4	5647	74	-0.15	0.1	1
12004971	71.3	0.3	6520	178	0.04	0.3	3
12068975	106.8	0.6	6004	102	-0.38	0.1	1
12314973	76.3	1.3	6215	89	0.28	0.1	1

The large frequency separations ($\Delta\nu$) are from this work, while the spectroscopic parameters (T_{eff} and [Fe/H]) have been found in the literature. The σ-columns give the 1σ uncertainties. For references for the spectroscopic values, see the papers listed in the Ref. column and references therein (K15 refers to Hans Kjeldsen et al. (in prep.)).

Supplementary References:

[1] Huber, D. *et al.* Fundamental Properties of Kepler Planet-candidate Host Stars using Asteroseismology. *ApJ* **767,** 127 (2013).

[2] Silva Aguirre, V. *et al.* Ages and fundamental properties of Kepler exoplanet host stars from asteroseismology. *MNRAS* **452,** 2127–2148 (2015).

[3] Huber, D. *et al.* Revised Stellar Properties of Kepler Targets for the Quarter 1-16 Transit Detection Run. *ApJS* **211,** 2 (2014).

[4] Chaplin, W. J. *et al.* Asteroseismic Fundamental Properties of Solar-type Stars Observed by the NASA Kepler Mission. *ApJS* **210,** 1 (2014).

[5] Buchhave, L. A. *et al.* An abundance of small exoplanets around stars with a wide range of metallicities. *Nature* **486,** 375–377 (2012).

[6] Marcy, G. W. *et al.* Masses, Radii, and Orbits of Small Kepler Planets: The Transition from Gaseous to Rocky Planets. *ApJS* **210,** 20 (2014).

[7] Rowe, J. F. *et al.* Validation of Kepler's Multiple Planet Candidates. III. Light Curve Analysis and Announcement of Hundreds of New Multi-planet Systems. *ApJ* **784,** 45 (2014).

[8] Everett, M. E. *et al.* High-Resolution Multi-Band Imaging for Validation and Characterization of Small Kepler Planets. *AJ* **149,** 55 (2015).

[9] Bakos, G. Á. *et al.* HAT-P-11b: A Super-Neptune Planet Transiting a Bright K Star in the Kepler Field. *ApJ* **710,** 1724–1745 (2010).
